# Four New Species of *Strobilomyces* (Boletaceae, Boletales) from Hainan Island, Tropical China

**DOI:** 10.3390/jof9121128

**Published:** 2023-11-22

**Authors:** Hui Deng, Yi Wang, Jin-Rui Lei, Zong-Zhu Chen, Zhi-Qun Liang, Nian-Kai Zeng

**Affiliations:** 1 Key Laboratory of Tropical Translational Medicine of Ministry of Education, School of Pharmacy, Hainan Medical University, Haikou 571199, China; 2 Ministry of Education Key Laboratory for Ecology of Tropical Islands, Key Laboratory of Tropical Animal and Plant Ecology of Hainan Province, College of Life Sciences, Hainan Normal University, Haikou 571158, China; 3 College of Science, Hainan University, Haikou 570228, China; 4 Hainan Academy of Forestry (Hainan Academy of Mangrove), Haikou 571100, China

**Keywords:** ectomycorrhizal fungi, molecular phylogeny, morphology, new taxa, taxonomy

## Abstract

*Strobilomyces*, one of the most noticeable genera of Boletaceae (Boletales), is both ecologically and economically important. Although many studies have focused on *Strobilomyces* in China, the diversity still remains incompletely understood. In the present study, several collections of *Strobilomyces* from Hainan Island, tropical China were studied based on morphology and molecular phylogenetic analyses. Four species are described as new, viz. *S*. *baozhengii*, *S*. *conicus*, *S*. *hainanensis*, and *S*. *pachycystidiatus*. Detailed descriptions, color photos of fresh basidiomata, and line drawings of microstructures of the four species are presented.

## 1. Introduction

*Strobilomyces* Berk., typified by *S. strobilaceus* (Scop.) Berk., is one of the most noticeable genera of Boletaceae (Boletales), which is characterized by its blackish, blackish-brown, reddish-brown or yellowish-brown pileus covered with scales, subglobose to elliptic basidiospores with reticulate, semi-reticulate, flat-roofed conical or echinate ornamentation, and an obvious reddening or blackening discoloration of tissues when bruised [1,2,3,4,5,6,7]. The genus is divided into two sections: *Echinati* L.H. Han, Zhu L. Yang & Ndolo Ebika and *Strobilomyces* Berk. Species of *Strobilomyces* are diverse; 80 names under the genus were recorded in the Index Fungorum database [http://www.Indexfungorum.org (accessed on 7 November 2023)] [7,8,9]. Interestingly, most species of the genus are reported from tropical and subtropical areas of Asia [1,6,7,8,10,11,12,13,14,15].

*Strobilomyces* plays an important role in maintaining the biodiversity of forest ecosystems, for species of the genus are usually symbiotic with many plants including Dipterocarpaceae, Fabaceae, Fagaceae, Myrtaceae, and Pinaceae [3,5,6,7]. Besides ecological value, the edible and medicinal values of the genus were also noted, as for example, *S. confusus* Singer, *S. glabriceps* W.F. Chiu, and *S. strobilaceus* (Scop.) Berk. were put on the Chinese edible mushroom list; *S. strobilaceus* was believed to have anticancer activity [16,17].

In China, species of *Strobilomyces* are also abundant. Currently, approximately 32 species of the genus have been uncovered [3,5,6,7,13,18,19,20,21,22]. Among them, most are from temperate and subtropical China, while a few species are from tropical regions of China. It is well known, tropical China especially Hainan Island is a biodiversity hotspot [23,24]. With more field investigations in the region, more species of the genus are expected to be revealed. During field investigations of Hainan Island, several collections of *Strobilomyces* were compiled; further morphology and molecular phylogenetic analyses confirm that these collections represent four novel species. They are described in an effort to further demonstrate the species diversity of Boletaceae in tropical China.

## 2. Materials and Methods

### 2.1. Morphological Studies

The fresh basidiomata were described and photographed in the field in daylight, then dried at 60 °C for 12 h. Dried specimens were deposited in the Fungal Herbarium of Hainan Medical University, Haikou City, Hainan Province of China (FHMU). Color codes comply with Kornerup and Wanscher [25]. The pileipellis sections were cut between the center and the margin of the pileus and the stipitipellis sections were taken from the middle part along the longitudinal axis of the stipe [26]. Samples were rehydrated using 5% KOH. All microscopic structures were drawn by freehand from rehydrated material. For basidiospores, the notation ‘n/m/p’ indicates ‘n’ basidiospores from ‘m’ basidiomata of ‘p’ collections. Dimensions of basidiospores are expressed as (a–)b–c(–d), where the range b–c represents a minimum of 90% of the measured values (5th to 95th percentile), and extreme values (a and d), whenever present (a < 5th percentile, d > 95th percentile), are expressed in parentheses. Q indicates the length/width ratio of basidiospores; Qm indicates the average Q of basidiospores and is given with standard deviation. The basidiospores were examined by Zeiss Sigma 300 scanning electron microscopy (SEM) using a small piece of hymenophore (2–5 mm diameter) from one dried specimen, which was sprayed with gold by ion sputtering. 

### 2.2. Molecular Procedures

Plant Genomic DNA Kit (CWBIO, Beijing, China) was used to extract total genomic DNA from materials dried with silica gel according to the manufacturer’s instructions. Primer pairs used for amplification were as follows: LR0R/LR5 [27,28] for nuc 28S rDNA D1–D2 domains (28S), ITS5/ITS4 [29] for nuc rDNA region encompassing the internal transcribed spacers 1 and 2 along with the 5.8S rDNA, the translation elongation factor 1-α gene (*tef1*) with EF1-F/EF1-R [30], and polymerase II second largest subunit gene (*rpb2*) with RPB2-B-F1/RPB2-B-R [19]. The PCR thermal cycling programs for 28S, ITS, *tef1*, and *rpb2* amplification were conducted as follows: 95 °C for 4 min, 35 cycles of 94 °C for 60 s, annealing at an appropriate temperature (50 °C for 28S and ITS, 53 °C for *tef1*, and 52 °C for *rpb2*) for 30 s, 72 °C for 4 min, and 72 °C for 7 min. The PCR products were purified (TIANGEN, Beijing, China) and then sequenced using a BigDye Terminator v3.1 Kit and an ABI 3730xl DNA Analyzer (Guangzhou Branch of BGI, China). Forward and reverse sequences were compiled with BioEdit v7.0.9.1 [31]. 

### 2.3. Dataset Assembly

A total of forty-five sequences (thirteen of 28S, thirteen of ITS, thirteen of *tef1*, and six of *rpb2*) from fifteen specimens were newly generated and edited sequences were deposited in GenBank, and accession numbers of 28S, ITS, *tef1*, and *rpb2* are provided in Table 1. The sequences were aligned with selected sequences from previous studies and GenBank (Table 1). *Anthracoporus holophaeus* (Corner) Yan C. Li & Zhu L. Yang (HKAS50508 and HKAS59407) was chosen as outgroup following Wu et al. [20]. Sequences of 28S, ITS, *tef1*, and *rpb2* were aligned, using MUSCLE (Edgar 2004) separately to test for phylogenetic conflict. There were no conflicts in the topologies of these trees, indicating that phylogenetic signals from different gene fragments were congruent. The sequences of the different genes were concatenated using Phyutility v. 2.2 for further analyses [32]. 

### 2.4. Phylogenetic Analyses

Maximum likelihood (ML) and Bayesian inference (BI) were used to reconstruct phylogenetic trees based on the combined nuclear dataset (28S + ITS + *tef1* + *rpb2*). ML analysis was conducted with the program RAxML 7.2.6 running 1000 replicates combined with an ML search. Bayesian analysis was conducted with MrBayes 3.1 using the Markov Chain Monte Carlo technique and parameters predetermined with MrModeltest 2.3 [41]. The best-fit likelihood models for the combined dataset were GTR + I + G, GTR + G, SYM + I + G, and K80 + I + G for 28S, ITS, *tef1*, and *rpb2*, respectively. Bayesian analysis of the combined nuclear dataset (28S + ITS + *tef1* + *rpb2*) was repeated for 30 million generations. Trees sampled from the first 25% of the generations were discarded as burn-in. The average SD of split frequencies was constrained to be below 0.01, and Bayesian posterior probabilities (PP) were then calculated for a majority consensus tree of the retained Bayesian trees.

## 3. Results

### 3.1. Molecular Data

**Figure 1:**
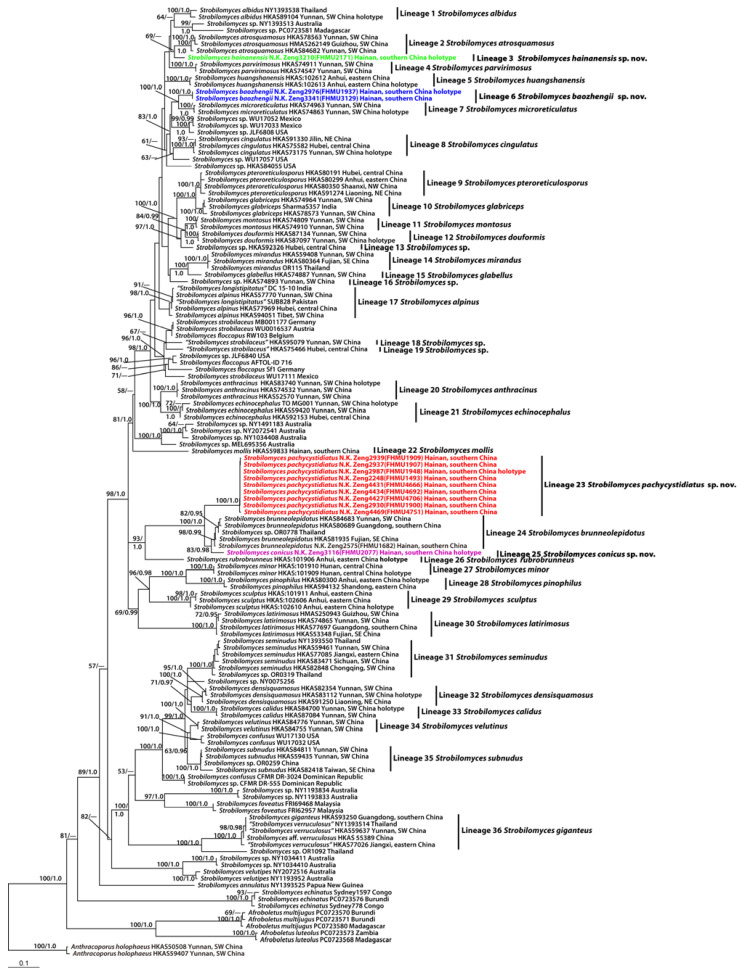
Phylogram inferred from a combined dataset (28S, ITS, *tef1*, and *rpb2*) using RAxML. RAxML likelihood bootstrap (BS ≥ 50%) and Bayesian posterior probabilities (PP ≥ 0.95) are indicated above or below the branches as RAxML BS/PP.

The combined dataset (28S + ITS + *tef1* + *rpb*2) included 140 taxa with 2679 nucleotide sites, and the alignment was deposited in TreeBASE (S30585) [https://treebase.org/treebase-web/home.html (accessed on 22 July 2023)]. Bayesian analyses resulted in identical topologies to the ML analysis, while statistical supports showed slight differences (Figure 1). The present molecular data indicated that the Chinese species of *Strobilomyces* were grouped into thirty-six independent lineages (Figure 1). A total of four new lineages were identified in this study (Lineages 3, 6, 23, and 25 of Figure 1). Lineage 3 comprised one collection (FHMU2171) from southern China; lineage 6, with strong statistical support (BS = 100%, PP = 1.0), included two new collections (FHMU1937 and FHMU3129) from southern China; lineage 23, with strong statistical support (BS = 100%, PP = 1.0), comprised nine collections (FHMU1493, FHMU1900, FHMU1907, FHMU1909, FHMU1948, FHMU4666, FHMU4692, FHMU4706 and FHMU4751) from southern China; lineage 25 comprised one collection (FHMU2077) also from southern China (Figure 1).

### 3.2. Taxonomy

**Figure 2:**
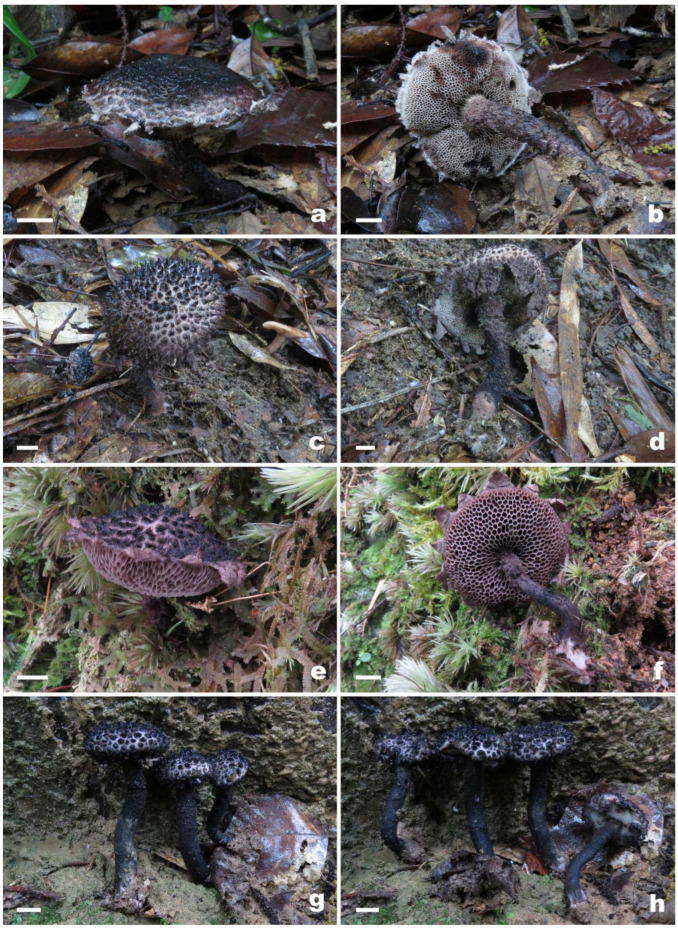
Basidiomata of *Strobilomyces* species. (**a**,**b**) *S*. *baozhengii* (FHMU1937, holotype); (**c**,**d**) *S*. *conicus* (FHMU2077, holotype); (**e**,**f**) *S*. *hainanensis* (FHMU2171, holotype); (**g**,**h**) *S*. *pachycystidiatus* (FHMU1948, holotype). Bars = 1 μm. Photos: N.K. Zeng.

**Figure 3:**
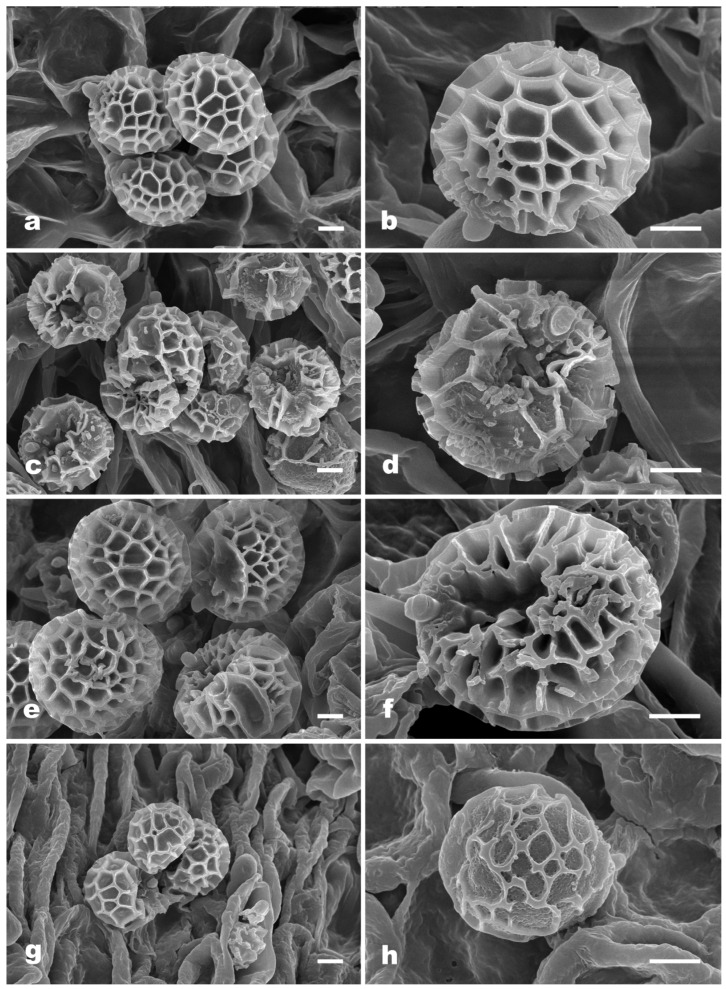
Scanning electron microscopy (SEM) photographs of basidiospores from *Strobilomyces* species. (**a**,**b**) *S*. *baozhengii* (FHMU1937, holotype); (**c**,**d**) *S*. *conicus* (FHMU2077, holotype); (**e**,**f**) *S*. *hainanensis* (FHMU2171, holotype); (**g**,**h**) *S*. *pachycystidiatus* (FHMU1948, holotype). Bars = 2 μm. Photos: H. Deng.

**Figure 4:**
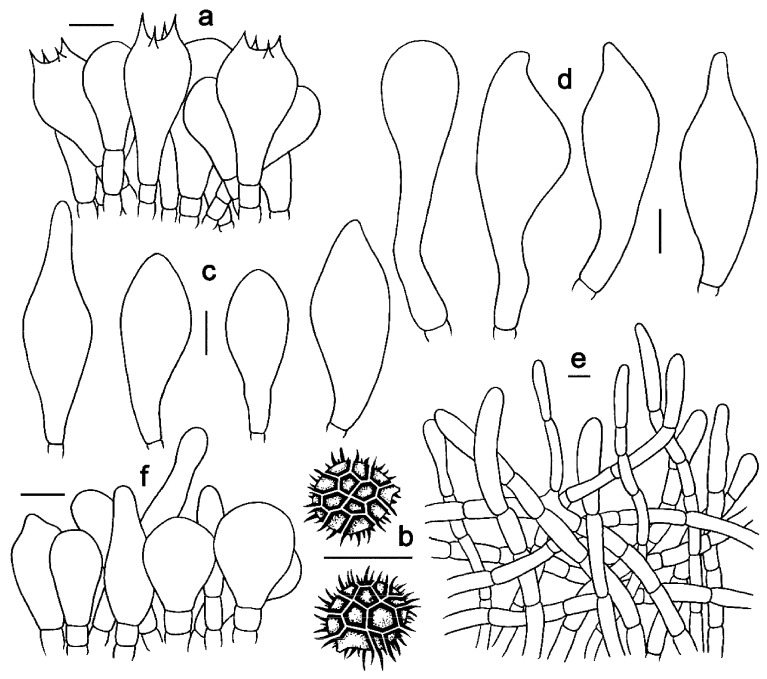
Microscopic features of *Strobilomyces baozhengii* (FHMU1937, holotype). (**a**) Basidia. (**b**) Basidiospores. (**c**) Cheilocystidia. (**d**) Pleurocystidia. (**e**) Pileipellis. (**f**) Stipitipellis. Bars = 10 μm. Drawings by Y. Wang.

***Strobilomyces baozhengii*** N.K. Zeng, Hui Deng, Yi Wang & Zhi Q. Liang, sp. nov. Figure 2a,b, Figure 3a,b and Figure 4.

**MycoBank no:** MB 850251

**Etymology:** “*baozhengii*” is named for Bao Zheng, a historic Chinese righteous officer, said to have a dark face.

**Diagnosis:** Differs from closest species of *Strobilomyces* by small basidioma (4.3–6 cm diameter), pileus densely covered with patch-like to appressed, light black to black scales, grey to black stipe covered with patch-like to appressed, blackish-brown scales or reticula, small hymenophoral pores (approximately 0.1 cm diameter), small- to medium-sized reticulate basidiospores (8–10.5 × 7–8.5μm), and its association with fagaceous trees.

**Holotype:** CHINA. Hainan Province: Yinggeling of Hainan Tropical Rainforest National Park, elev. 550 m, 26 May 2017, N.K. Zeng2976 (FHMU1937).

**Description:** Pileus 4.3–6 cm diameter, subhemispherical when young, then convex to applanate; surface dirty white, densely covered with light black (11C1) to black (11F1), patch-like to appressed scales; margin usually appendiculate with thick and triangular or irregular lacy veil remnants concolorous with pileal surface; context 0.7–1.5 cm in thickness in the center of the pileus, white (2A1) to greyish-white (7B1), turning red (9E5) slowly then black (9F1) quickly when injured. Hymenophore tubulate, slightly depressed around the apex of the stipe; pores angular, approximately 0.1 cm diameter, tubes 0.6–0.7 cm long, greyish-white (2A1) to greyish-black (7D1), turning reddish (9E5) then black (9F1) when injured. Stipe 4.5–6 × 1–1.5 cm, central, subcylindrical, solid, grey (7B1) to black (7F1); surface covered with patch-like to appressed blackish-brown (7E1) scales or reticula; context greyish-white (11F1), turning reddish (9E5) or reddish-brown (9E7) then black (9F1) when injured; annulus at the apex but not very obvious; basal mycelium greyish-white (7A1). Odor indistinct.

Basidia 24–38 × 12–19.5 μm, subclavate or clavate, thin-walled, sometimes slightly thick-walled (up to 0.5 μm), colorless, pale yellowish-brown to yellowish-brown in KOH; sterigmata 2–7 μm in length. Basidiospores [60/3/2] 11–13.5 × (10–)10.5–12(–14) μm, Q = (0.96–)1.00–1.18(–1.20), Qm = 1.07 ± 0.06 (including ornamentation); [40/2/2] (7–)8–10.5(–11) × 7–8.5 μm, Q = 1.00–1.31 (–1.47), Qm = 1.16 ± 0.10 (excluding ornamentation), spherical to broad ellipsoid, yellowish-brown to darkish-brown in KOH, reticulate, with meshes 1–5 μm high. Hymenophoral trama boletoid, composed of colorless to pale yellow in KOH, 4–15 μm wide, thin- to slightly thick-walled (up to 0.5 μm) hyphae. Cheilocystidia 37–55 × 13–20 μm, fusiform or subfusiform, thin- to slightly thick-walled (up to 1 μm), colorless, pale yellowish-brown to yellowish-brown in KOH. Pleurocystidia 50–65 × 16–21 μm, fusiform, subfusiform or subclavate, thin- to slightly thick-walled (up to 0.5 μm), yellowish-brown in KOH. Pileipellis an intricate trichodermium 150–280 μm thick, composed of 5–14 μm wide, colorless to yellowish-brown in KOH, thin- to slightly thick-walled (up to 1 μm) hyphae; terminal cells 24–57 × 5–10 μm, clavate or subcylindrical, with obtuse apex. Pileal trama composed of hyphae 4–16 μm in diameter, thin- to slightly thick-walled (up to 0.5 μm), yellow in KOH. Stipitipellis a trichoderm-like structure 60–230 μm thick, composed of thin-walled, colorless, pale yellowish-brown to yellowish-brown in KOH, emergent hyphae with clavate or subglobose terminal cells (19–44 × 6–22 μm). Stipe trama composed of parallel hyphae 4–12 μm wide, cylindrical, thin- to slightly thick-walled (up to 0.5 μm), colorless or pale yellowish-brown in KOH. Clamp connections absent in all tissues.

**Additional specimen examined:** CHINA. Hainan Province: Yinggeling of Hainan Tropical Rainforest National Park, elev. 550 m, 6 May 2018, N.K. Zeng3341 (FHMU3129).

**Habitat:** Scattered on the ground in forests dominated by fagaceous trees.

**Known distribution:** Southern China (Hainan Province).

**Notes:** *Strobilomyces baozhengii* is phylogenetically related to *S*. *microreticulatus* Li H. Han & Zhu L. Yang (Figure 1). However, *S*. *microreticulatus*, originally described from Yunnan Province, southwestern China, differs by a stipe with grey-white and grey-black flosses above and below the annulus, and larger basidiospores (9–11 × 7–8 μm) with lower meshes (1 μm high) [7]. Morphologically, *S*. *baozhengii* is similar to Chinese *S*. *echinocephalus* Gelardi & Vizzini and *S*. *parvirimosus* J.Z. Ying, Malaysian *S*. *mollis* Corner, and Indian *S*. *nigricans* Berk. However, *S*. *echinocephalus* has a pileus covered with erect scales and smaller basidiospores measuring 8–11.5 × 6.8–9.9 μm (including ornamentation) [8]; *S*. *parvirimosus* has a pileus covered with erect pyramidal scales and basidiospores with lower meshes (up to 1 μm) [7,42]; *S*. *mollis* has a blackish-brown to vinaceous pileus with erect conical scales and smaller basidiospores (7.5–9.5 × 6.5–8 μm) [1,7]; *S*. *nigricans* has larger basidiospores (9.5–12 × 7.5–9.5 μm) and grows in association with *Abies* spp. [7,43].

**Figure 5:**
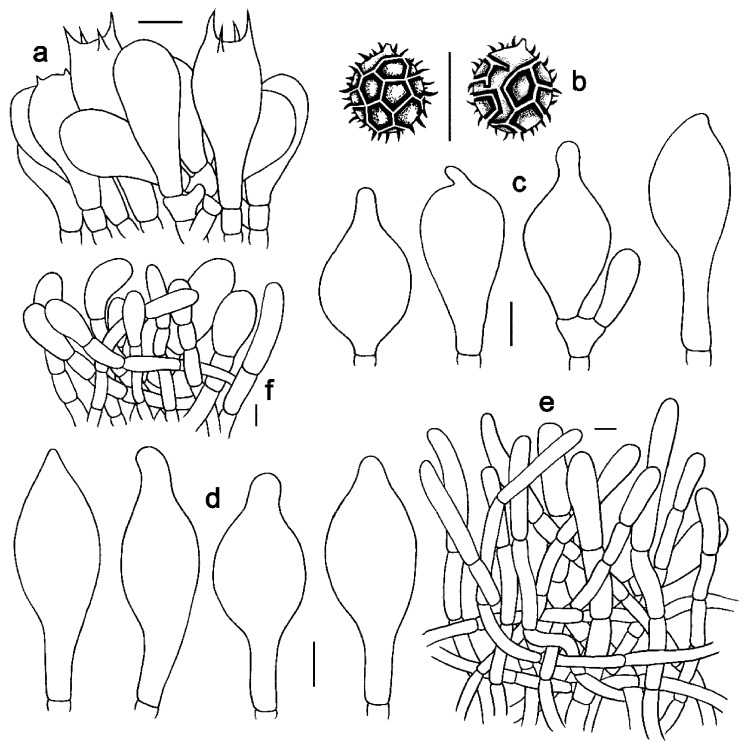
Microscopic features of *Strobilomyces conicus* (FHMU2077, holotype). (**a**) Basidia. (**b**) Basidiospores. (**c**) Cheilocystidia. (**d**) Pleurocystidia. (**e**) Pileipellis. (**f**) Stipitipellis. Bars = 10 μm. Drawings by Y. Wang.

***Strobilomyces conicus*** N.K. Zeng, Hui Deng, Yi Wang & Zhi Q. Liang, sp. nov. Figure 2c,d, Figure 3c,d and Figure 5.

**MycoBank no:** MB 850252

**Etymology:** “*conicus*” refers to the conical scales on pileus.

**Diagnosis:** Differs from closest species of *Strobilomyces* by medium-sized basidioma (approximately 6.5 cm diameter), pileus densely covered with brownish-black to black, erect conical scales or villi, small hymenophoral pores (0.05–0.1 cm diameter), small incomplete reticulate to reticulate basidiospores (7.5–9 × 7–7.5 μm), and its association with fagaceous trees.

**Holotype:** CHINA. Hainan Province: Limushan of Hainan Tropical Rainforest National Park, elev. 650 m, 26 July 2017, N.K. Zeng3116 (FHMU2077). 

**Description:** Pileus approximately 6.5 cm diameter, subhemispherical when young, then convex to applanate; surface dirty white, densely covered with brownish-black (7D3) to black (7F1), erect conical scales or villi; margin usually appendiculate with thick and triangular remnants concolorous with pileal surface; context approximately 1 cm in thickness in the center of the pileus, greyish-white (2B2), immediately turning reddish-brown (7D5) then black (7F1) when injured. Hymenophore tubulate, adnate; pores angular, 0.05–0.1 cm diameter, tubes 0.2–0.7 cm long, white (2A1) or greyish-white (2B2), turning reddish-brown (7E4) slowly then black (7F1) quickly when injured. Stipe 6.5 × 0.8–1 cm, central, subcylindrical, solid; surface covered with black (7F1) scales or villi; context greyish-black (5E1), unchanging in color when injured; basal mycelium white (2A1). Odor indistinct. 

Basidia 27–43 × 11–20 μm, subclavate or clavate, thin-walled, colorless to pale yellowish-brown in KOH; sterigmata 2–6 μm in length. Basidiospores [20/1/1] (9–)10–11(–12) × (9–)10–11(–11.5) μm, Q = 1.00–1.10, Qm = 1.01 ± 0.03 (including ornamentation); [20/1/1] 7.5–9 × 7–7.5(–8.5) μm, Q = (1.00–)1.06–1.20(–1.21), Qm = 1.11 ± 0.06 (excluding ornamentation), spherical to broad ellipsoid, yellowish-brown to darkish-brown in KOH, incomplete reticulate to reticulate, with meshes 1–3 μm high. Hymenophoral trama boletoid; composed of colorless to yellowish-brown in KOH, 5–16 μm wide, thin-walled hyphae. Cheilocystidia 36–54 × 15–23 μm, ventricose, fusiform or subfusiform, thin- to slightly thick-walled (up to 1 μm), colorless to pale yellowish-brown in KOH. Pleurocystidia 41–61 × 16–25 μm, ventricose, fusiform or subfusiform, thin- to slightly thick-walled (up to 1 μm), colorless to yellowish-brown in KOH. Pileipellis an intricate trichodermium 160–350 μm thick, composed of 5–17 μm wide, yellow in KOH, thin- to slightly thick-walled (up to 1 μm) hyphae; terminal cells 27–70 × 6–15 μm, clavate or subclavate, with obtuse apex. Pileal trama composed of hyphae 3–16 μm in diameter, thin- to slightly thick-walled (up to 0.5 μm), colorless in KOH. Stipitipellis a trichoderm-like structure 80–240 μm thick, composed of thin- to thick-walled (up to 2 μm), yellowish-brown in KOH, emergent hyphae with spherality, pyriform, clavate or subclavate terminal cells (21–47 × 6–20 μm). Stipe trama composed of parallel hyphae 6–28 μm wide, cylindrical, thin- to slightly thick-walled (up to 0.5 μm), and colorless to pale yellowish-brown in KOH. Clamp connections absent in all tissues.

**Habitat:** Scattered on the ground in forests dominated by fagaceous trees. 

**Known distribution:** Southern China (Hainan Province).

**Notes:** Phylogenetically, *S*. *conicus* is related to *S*. *brunneolepidotus* Har. Takah. & Taneyama and *S*. *pachycystidiatus* N.K. Zeng, Hui Deng, Yi Wang & Zhi Q. Liang (Figure 1). However, *S*. *brunneolepidotus*, originally described from Japan, differs by its reddish-brown scales on pileus [7,44]; *S*. *pachycystidiatus*, also described from tropical China, is distinguished by its larger hymenophoral pores (0.1–0.5 cm diameter), thicker cystidia (thick to 2–3 μm), and basidiospores with lower meshes (0.5–2.5 μm high). Morphologically, *S*. *conicus* is similar to *S*. *anthracinus* Li H. Han, J. Xu & Zhu L. Yang and *S*. *calidus* Li H. Han, J. Xu & Zhu L. Yang, *S*. *echinocephalus*, *S*. *microreticulatus*, *S*. *mollis*, *S*. *nigricans*, and *S*. *parvirimosus*. However, *S*. *anthracinus* has a stipe with thin fluffy flosses and completely reticulate basidiospores with lower meshes (1–1.5 μm high) [7]; *S*. *calidus* has a larger basidioma, larger hymenophoral pores (0.1–0.2 cm diameter), larger basidiospores with lower meshes (0.5–1 μm high) [7]; *S*. *echinocephalus* has a smaller basidioma and smaller basidiospores measuring 8–11.5 × 6.8–9.9 μm (including ornamentation) [8]; *S*. *microreticulatus* has a smaller basidioma, a stipe with grey-white and grey-black flosses above and below annulus, and larger basidiospores (9–11 × 7–8 μm) with lower meshes (1 μm high) [7]; *S*. *mollis* has larger hymenophoral pores (1–1.5 mm diameter) and completely reticulate basidiospores [1,7]; *S*. *nigricans* has larger basidiospores (9.5–12 × 7.5–9.5 μm) and associates with *Abies* spp. [7,43]; *S*. *parvirimosus* has larger basidiospores (8–10 × 6.5–8 μm) with lower meshes (1 μm high) [7,42].

**Figure 6:**
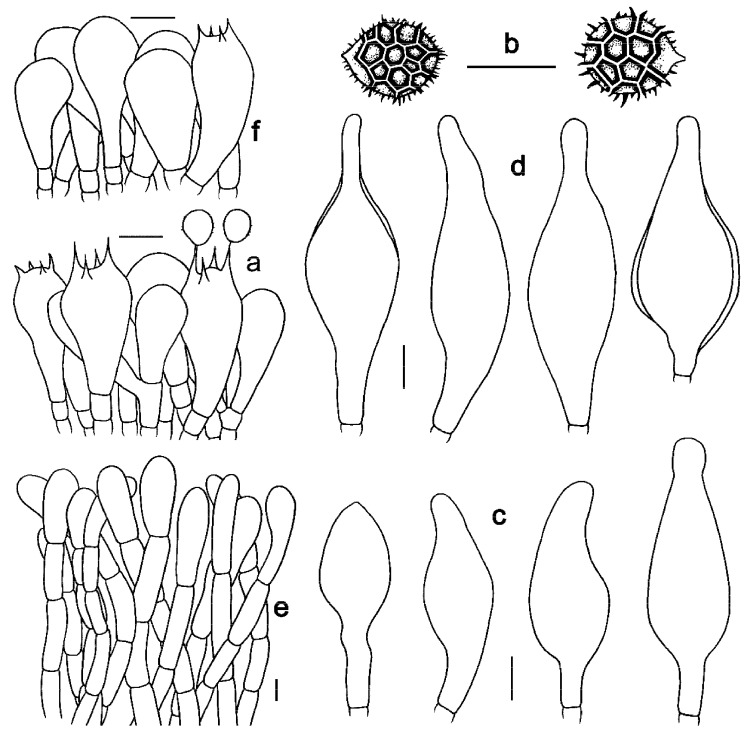
Microscopic features of *Strobilomyces hainanensis* (FHMU2171, holotype). (**a**) Basidia. (**b**) Basidiospores. (**c**) Cheilocystidia. (**d**) Pleurocystidia. (**e**) Pileipellis. (**f**) Stipitipellis. Bars = 10 μm. Drawings by Y. Wang.

***Strobilomyces hainanensis*** N.K. Zeng, Hui Deng, Yi Wang & Zhi Q. Liang, sp. nov. Figure 2e,f, Figure 3e,f and Figure 6.

**MycoBank no:** MB 850253.

**Etymology:** “*hainanensis*” refers to Hainan Province, China, holotype locality.

**Diagnosis:** Differs from closest species of *Strobilomyces* by smaller basidioma (approximately 4 cm diameter), pileus densely covered with black, erect conical to pyramidal scales, stipe covered with black fluffy flosses, small hymenophoral pores (approximately 0.1 cm diameter), medium-sized reticulate basidiospores (9–11.5 × 8–10 μm), and its association with fagaceous trees. 

**Holotype:** CHINA. Hainan Province: Jianfengling of Hainan Tropical Rainforest National Park, elev. 950 m, 31 July 2017, N.K. Zeng3210 (FHMU2171). 

**Description:** Pileus approximately 4 cm diameter, subhemispherical when young, then convex to applanate; surface dirty white, densely covered with black (11F1), conical to pyramidal scales; appendiculate thick and triangular veil remnants along the margin, dark reddish-brown (11C3); context approximately 0.3 cm in thickness in the center of the pileus, turning reddish (9E5) then black (9F1) when injured. Hymenophore tubulate, slightly depressed around the apex of the stipe; pores angular, approximately 0.1 cm diameter, tubes approximately 0.6 cm long, greyish-white (2A1) to greyish-black (7D1), turning reddish (9E5) then black (9F1) when injured. Stipe 4–5 × 0.5–0.7 cm, central, subcylindrical or slightly enlarged at base, solid; surface covered with black (12F1) fluffy flosses, context greyish-white (11F1), turning reddish (9E5) or reddish-brown (9E7) then black (9F1) when injured; basal mycelium white (1A1). Odor indistinct.

Basidia 23–40 × 11–20 μm, clavate, thin-walled, colorless to yellowish-brown in KOH; sterigmata 4–6 μm in length. Basidiospores [20/1/1] (10.5)11–13(–14) × 10–13 μm, Q = 1.00–1.14(–1.30), Qm = 1.08 ± 0.07 (including ornamentation); [20/1/1] (8.5–)9–11.5(–12) × 8–10 μm, Q = 1.06–1.24 (–1.25), Qm = 1.15 ± 0.06 (excluding ornamentation), spherical to broad ellipsoid, yellowish-brown to darkish-brown in KOH, reticulate, with meshes 1–4 μm high. Hymenophoral trama boletoid; composed of colorless to yellowish-brown in KOH, 3–14 μm wide, thin- to slightly thick-walled (up to 1 μm) hyphae. Cheilocystidia 21–63 × 12–20 μm, abundant, lageniform, ventricose or subfusiform, thin- to slightly thick-walled (up to 1 μm), yellowish-brown in KOH. Pleurocystidia 36.5–68 × 12.5–28 μm, abundant, fusiform or subfusiform, thin- to slightly thick-walled (up to 1 μm), yellowish-brown to darkish-brown in KOH. Pileipellis a trichoderm approximately 300 μm thick, composed of more or less vertically arranged, 7–19 μm wide, pale yellowish-brown to yellowish-brown in KOH, thin- to slightly thick-walled (up to 1 μm) hyphae; terminal cells 25–68 × 10–17 μm, clavate or subcylindrical, with obtuse apex. Pileal trama composed of hyphae 4–31 μm in diameter, thin- to slightly thick-walled (up to 0.5 μm), colorless or pale yellow in KOH. Stipitipellis a trichoderm-like structure 50–100 μm thick, composed of thin- to slightly thick-walled (up to 0.5 μm), yellowish-brown in KOH, emergent hyphae with subclavate or clavate terminal cells (25–35 × 12–18 μm). Stipe trama composed of parallel hyphae 4–12 μm wide, cylindrical, thin- to slightly thick-walled (up to 0.5 μm), and colorless to yellowish-brown in KOH. Clamp connections absent in all tissues.

**Habitat:** Solitary on the ground in forests dominated by *Lithocarpus* spp., *Leucobryum javense* (Brid.) Mitt., and *Calypogeia* sp.

**Known distribution:** Southern China (Hainan Province).

**Notes:** Our new species *S. hainanensis* formed a separate species-level branch at the phylogenetic tree (Figure 1), indicating that it is in an independent phylogenetic position. Morphologically, *S*. *hainanensis* is similar to *S*. *echinocephalus*, *S*. *microreticulatus*, *S*. *mollis*, *S*. *nigricans*, and *S*. *parvirimosus*. However, *S*. *echinocephalus* has a larger basidioma and smaller basidiospores measuring 8–11.5 × 6.8–9.9 μm (including ornamentation) [8]; *S*. *microreticulatus* has a pileus with dirty white scales on periphery, a stipe with grey-white flosses above annulus, and narrower basidiospores (9–11 × 7–8 μm) [7]; *S*. *mollis* has smaller basidiospores (7.5–9.5 × 6.5–8 μm) [1,7]; *S*. *nigricans* is associated with *Abies* spp. in subalpine areas in northern India [7,43]; *S*. *parvirimosus* has smaller basidiospores (8–10 × 6.5–8 μm) with lower meshes (up to 1 μm) [7,42].

**Figure 7:**
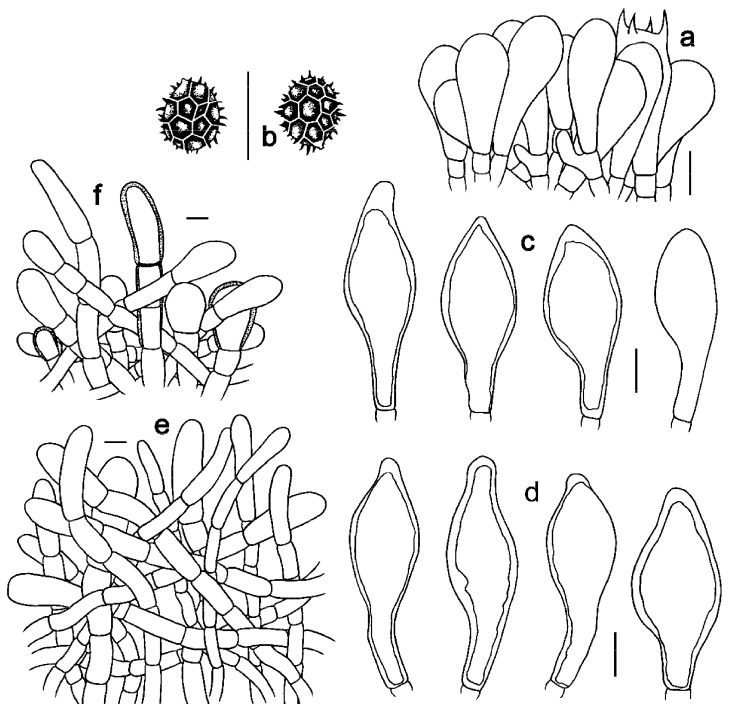
Microscopic features of *Strobilomyces pachycystidiatus* (FHMU1948, holotype). (**a**) Basidia. (**b**) Basidiospores. (**c**) Cheilocystidia. (**d**) Pleurocystidia. (**e**) Pileipellis. (**f**) Stipitipellis. Bars = 10 μm. Drawings by Y. Wang.

***Strobilomyces pachycystidiatus*** N.K. Zeng, Hui Deng, Yi Wang & Zhi Q. Liang, sp. nov. Figure 2g,h, Figure 3g,h and Figure 7.

**MycoBank no:** MB 850254

**Etymology:** Latin “*pachy*” meaning thick, “*cystidiatus*” meaning cystidium, “*pachycystidiatus*” refers to the thick-walled cystidia.

**Diagnosis:** Differs from closest species of *Strobilomyces* by tiny to medium basidioma (2.7–7.5 cm diam), pileus densely covered with blackish-brown to black conical scales, stipe covered with darkish-brown to black scales or fluffy flosses, large hymenophoral pores (0.1–0.5 cm diameter), small- to medium-sized reticulate basidiospores (6.5–11 × 4.5–9 μm), thick-walled (2–3 μm) cystidia, and its association with fagaceous trees.

**Holotype:** CHINA. Hainan Province: Yinggeling of Hainan Tropical Rainforest National Park, elev. 550 m, 26 July 2017, N.K. Zeng2987 (FHMU1948).

**Description:** Pileus 2.7–7.5 cm diameter, subhemispherical when young, then convex to applanate; surface dirty white, densely covered with blackish-brown (7F1) to black (11F1), conical scales; margin usually appendiculate with thick and triangular or irregular lacy veil remnants concolorous with pileal surface; context 0.6–1.4 cm in thickness in the center of the pileus, white (2A1) to greyish-white (5A1), turning reddish-brown (5B3) then black (7F1) when injured. Hymenophore tubulate, slightly depressed around the apex of the stipe; pores angular, 0.1–0.5 cm diameter, tubes 0.4–1.1 cm long, white (3A1) or greyish-white (5A1), turning reddish-brown (5B3) then black (7F1) when injured. Stipe 4.6–9 × 0.6–1.4 cm, central, subcylindrical, solid; surface densely covered with darkish-brown (7E1) to black (7F1) scales or fluffy flosses; context white (2A1) to greyish-white (5A1), turning reddish-brown (5B3) then black (7F1) when injured; basal mycelium white (2A1) or greyish-white (7A1). Odor indistinct.

Basidia 19–33 × 10–15 μm, subclavate or clavate, thin- to slightly thick-walled (up to 0.5 μm), colorless to pale yellowish-brown in KOH; sterigmata 2–6 μm in length. Basidiospores [300/15/9] 8.5–11(–12) × (7–)8–11 μm, Q = 1.00–1.13(–1.21), Qm = 1.06 ± 0.05 (including ornamentation); [300/15/9] 7–9 × 6–8(–8.5) μm, Q = 1.00–1.33(–1.42), Qm = 1.18 ± 0.11 (excluding ornamentation), spherical to broad ellipsoid, yellowish-brown to darkish-brown in KOH, reticulate, with meshes 0.5–2.5 μm high. Hymenophoral trama boletoid; composed of colorless to yellowish-brown in KOH, 4–20 μm wide, thin- to slightly thick-walled (up to 0.5 μm) hyphae. Cheilocystidia 30–54 × 13–20 μm, abundant, ventricose, fusiform or subfusiform, thin- to thick-walled 5 (up to 2 μm), colorless to yellowish-brown in KOH. Pleurocystidia 35–57 × 13–20 μm, abundant, ventricose, fusiform or subfusiform, thin- to thick-walled (up to 3 μm), yellowish-brown in KOH. Pileipellis an intricate trichodermium 130–700 μm thick, composed of 5–21 μm wide, pale yellowish-brown to yellowish-brown in KOH, thin-walled hyphae; terminal cells 31–71 × 7–19 μm, pyriform, clavate or subclavate, with obtuse apex. Pileal trama composed of hyphae 5–17 μm in diameter, thin- to slightly thick-walled (up to 0.5 μm), colorless to yellowish-brown in KOH. Stipitipellis a trichoderm-like structure 50–400 μm thick, composed of thin- to thick-walled (up to 2 μm), yellowish-brown in KOH, emergent hyphae with spherality, clavate or subclavate terminal cells (15–52 × 10–30 μm). Stipe trama composed of longitudinally arranged, parallel hyphae 3–15 μm wide, cylindrical, thin- to slightly thick-walled (up to 0.5 μm), colorless, pale yellowish-brown to darkish-brown in KOH. Clamp connections absent in all tissues.

**Additional specimens examined:** CHINA. Hainan Province: Yinggeling of Hainan Tropical Rainforest National Park, elev. 550 m, 30 July 2015, N.K. Zeng2248 (FHMU1493); same location, 9 September 2016, N.K. Zeng2930 (FHMU1900); same location, 10 September 2016, N.K. Zeng2937, 2939 (FHMU1907, 1909); same location, 3 July 2020, N.K. Zeng4427, 4431, 4434, 4469 (FHMU4706, 4666, 4692, 4683, 4751).

**Habitat:** Solitary or scattered on the ground in forests dominated by fagaceous trees. 

**Known distribution:** Southern China (Hainan Province).

**Notes:** *Strobilomyces pachycystidiatus* is phylogenetically closely related to *S*. *brunneolepidotus*. However, *S*. *brunneolepidotus* has a basidioma with reddish-brown scales and thin-walled cystidia [7,44]. Morphologically, *S*. *pachycystidiatus* is similar to *S*. *echinocephalus*, *S*. *microreticulatus*, *S*. *mollis*, *S*. *nigricans*, and *S*. *parvirimosus*. However, *S*. *echinocephalus* has a stipe densely covered by cottony to woolly floccules, larger basidiospores measuring 8–11.5 × 6.8–9.9 μm (including ornamentation) and smaller hymenophoral pores (approximately 0.1 cm diameter) [8]; *S*. *microreticulatus* has a smaller basidioma, a stipe with grey-white flosses above annulus, larger basidiospores (9–11 × 7–8 μm), and smaller hymenophoral pores (0.05–0.1cm diameter) [7]; *S*. *mollis* has a stipe with blackish-brown to vinaceous, thin fluffy flosses, basidiospores with higher meshes (2–3.5 μm high), and smaller hymenophoral pores (0.1–0.15 cm diameter) [1,7]; *S*. *nigricans* has larger basidiospores (9.5–12 × 7.5–9.5 μm), its association with *Abies* spp., and its distribution in subalpine areas in northern India [7,43]; *S*. *parvirimosus* has smaller basidiospores (8–10 × 6.5–8 μm) with lower meshes (1 μm high), and smaller hymenophoral pores (0.05–0.1 diameter) [7,42]. 

## 4. Discussion

Although the diagnosis of *Strobilomyces* is relatively easy, species within the genus are difficult to identify because of an absence of molecular data as well as morphological convergence documented in this group in previous studies [11,12]. With the development of molecular phylogenetic analyses, many previously described taxa have been re-evaluated, which enhanced our understanding of *Strobilomyces* diversity worldwide [7,8,14]. For example, *S*. *confusus* and *S*. *strobilaceus*, two taxa originally described from North America and Europe, respectively [45], were previously thought to be widely distributed species. However, recent studies have indicated that *S*. *confusus* and *S*. *strobilaceus* actually represent species complexes [7,8,14]. Our molecular data also showed that specimens identified as *S*. *confusus* or *S*. *strobilaceus* were present in different parts of the tree (Figure 1). 

High species diversity of *Strobilomyces* in China was revealed in the present study, and thirty-six lineages of *Strobilomyces* were identified (Figure 1). Four (lineages 3, 6, 23, and 25) were described as new species: *S*. *hainanensis*, *S*. *baozhengii*, *S*. *pachycystidiatus,* and *S*. *conicus;* twenty-eight (lineages 1, 2, 4, 5, 7, 8, 9, 10, 11, 12, 14, 15, 17, 20, 21, 22, 24, 26, 27, 28, 29, 30, 31, 32, 33, 34, 35, and 36) represent previously described taxa (Table 2); four (lineage 13, 16, 18, and 19) are awaiting further identification (Figure 1). Together with previously described species of *Strobilomyces* in China (Table 2), our new species *S*. *baozhengii*, *S*. *conicus*, *S*. *hainanensis*, and *S*. *pachycystidiatus* are all members of sect. *Strobilomyces* (Figure 1). Interestingly, species of sect. *Echinati* have not been uncovered in China hitherto.

Recent phylogenetic studies have provided new perspectives into the phylogeny and geography of *Strobilomyces* [5,7]. Besides revealing four new species of *Strobilomyces*, our molecular data have also contributed to other knowledge of this group (Figure 1). The molecular data indicated the affinities of *Strobilomyces* species between China and Europe, with two Chinese collections both labeled as *S*. *strobilaceus* (lineages 18 and 19) being related to the European taxa (Figure 1). Moreover, we also noted that some Chinese species are closely related to the North America taxa; for example, *S*. *velutinus* (lineage 34) from southwestern China is affiliated with one taxon labeled as *S*. *confusus* from USA. Interestingly, the Asian *S. albidus* is related to two unidentified collections from Australia and Africa, respectively (Figure 1). In addition, we noted there are several common taxa from China and Thailand, i.e., *S*. *albidus*, *S*. *brunneolepidotus, S*. *giganteus*, *S*. *mirandus*, and *S. seminudus* (Figure 1). *Strobilomyces alpinus* is widely distributed, occurring in the south of China, Pakistan, and India (Figure 1). The affinities of *S*. *glabriceps* between the south of China and India are evident (Figure 1).

## 5. Conclusions

Although *Strobilomyces* is widely distributed in the world, the diversity of this genus has not been completely resolved. In this work, four new species of *Strobilomyces*, viz. *S. baozhengii*, *S. conicus*, *S. hainanensis*, and *S. pachycystidiatus* are described based on morphological and molecular phylogenetic analysis, which provides us with further understanding of this genus diversity in tropical China. In the future, we hope that more and more species of the genus will be uncovered.

**Key to the species of *Strobilomyces* in China.**

1. Pileus with blackish-brown, reddish-brown, yellowish-brown, brown or golden-tawny scales21. Pileus with black, greyish-black, grey or dirty white scales172. Pileus with golden-tawny to golden-orange scales; basidiospores smaller measuring 6–8 × 5.5–7 μm*S*. *mirandus*2. Pileus with blackish-brown, reddish-brown, brown or yellowish-brown scales; basidiospores larger, more than 7 μm in length 33. Pileus smaller (up to 4 cm), with yellowish-brown scales*S*. *minor*3. Pileus larger (up to 12 cm), with blackish-brown, reddish-brown or brown scales44. Pileus with reddish-brown or brown scales54. Pileus with blackish-brown scales95. Stipe with dirty white to light reddish-brown thin flosses; hymenophoral pores smaller, 0.05–0.1 cm diameter
*S. glabellus*
5. Stipe with reddish-brown, brown, dark brown to blackish-brown fluffy flosses; hymenophoral pores larger, 0.1–0.3 cm diameter66. Pileus with reddish-brown to vinaceous brown on the lower part to dark brown to blackish-brown on the upper part, crowded, more or less erect pyramidal scales; stipe with dark brown to blackish-brown fluffy flosses
*S. atrosquamosus*
6. Pileus with reddish-brown or brown, scattered, erect conical scales; stipe with concolorous fluffy flosses and conical scales7 7. Hymenophoral pores smaller, 0.05–0.1 cm diameter; stipe with light grayish-brown to chocolate fluff on the upper part and reddish-brown to chocolate fluff on the lower, basidiospores larger measuring 8–10 × 7–8 μm*S*. *rubrobrunneus*7. Hymenophoral pores larger, 0.1–0.3 cm diameter; stipe with brown or reddish fluffy flosses or scales, basidiospores smaller, less than 9 μm in length88. Scales on pileus brown, without reddish tinge; stipe surface reticulate with shallow and elongate meshes on the upper part, covered with clustered tomentose scales on the lower part; basidiospores smaller measuring 7–8 × 6.5–7 μm*S*. *sculptus*8. Scales on pileus with reddish tinge; stipe with concolorous thick fluffy flosses and erect conical scales; basidiospores larger measuring 7.5–9 × 6.5–8 μm
*S. brunneolepidotus*
9. Pileus with more or less erect conical to pyramidal scales109. Pileus with patch-like to appressed scales or flosses1510. Basidiospores with complete reticula; hymenophoral pores larger, 0.05–0.5 cm diameter1110. Basidiospores with incomplete reticula; hymenophoral pores smaller, 0.05–0.1 cm diameter*S*. *conicus*11. Hymenophoral pores larger, 0.1–0.5 cm diameter; cystidia thick-walled (up to 3 μm); tropical distribution
*S. pachycystidiatus*
11. Hymenophoral pores smaller, 0.05–0.15 cm diameter; cystidia thin-walled, less than 1 μm; temperate, tropical or subtropical distribution1212. Stipe with blackish-brown thin fluffy flosses 1312. Stipe with sooty-brown to blackish, grey to dirty white or greyish-black flosses1413. Pileus with blackish-brown to vinaceous, soft erect conical scales; basidiospores with larger meshes (2–3.5 μm diameter)*S*. *mollis*13. Pileus with black-brown, more or less erect pyramidal scales; basidiospores with smaller meshes (1–2 μm diameter) *S*. *parvirimosus*14. Pileus with sooty-brown to blackish scales; stipe without an annulus, with cottony or woolly, sooty-brown to blackish floccules*S*. *echinocephalus*14. Pileus with dirty white to light blackish-brown scales; stipe with an annulus, with grey to dirty white on the upper part and greyish-black flosses on the lower part*S*. *microreticulatus*15. Hymenophoral pores larger, 0.1–0.2 mm diameter; basidiospores larger measuring 11.5–14 × 9.5–11 μm, with larger meshes (2–4 μm diameter)*S*. *alpinus*15. Hymenophoral pores smaller, 0.05–0.1 mm diameter; basidiospores smaller, less than 11 μm in length, with smaller meshes (1–3 μm diameter)1616. Pileus with blackish-brown at apex and light brown to dirty white at base, scales or flosses; stipe with an annulus, with greyish-white fluffy flosses on the upper part and dark blackish-brown on the lower arranged in spiral; basidiospores larger measuring 9–11 × 7–8.5 μm; subtropical to temperate distribution*S*. *cingulatus*16. Pileus with blackish-brown to dark chocolate scales or flosses; stipe without an annulus, with dirty white thin flosses evenly distributed; basidiospores smaller measuring 7–9 × 6–7 μm; tropical distribution *S*. *albidus*17. Pileus with black scales1817. Pileus with greyish-black, grey to dirty white scales2418. Pileus with more or less erect conical to pyramidal scales1918. Pileus with patch-like to appressed scales or flosses2319. Basidiospores with complete reticula2019. Basidiospores with incomplete reticula2220. Pileus larger (up to 9.7 cm); stipe with thick flosses arranged in spiral
*S. douformis*
20. Pileus smaller (up to 5 cm); stipe with thin flosses evenly distributed2121. Pileus with charcoal black, smaller, more or less erect pyramidal scales; basidiospores smaller measuring 8.5–10 × 6.5–8(–9) μm; subtropical distribution
*S. anthracinus*
21. Pileus with black erect conical to pyramidal scales; basidiospores larger measuring 9–11.5 × 8–10 μm; tropical distribution
*S. hainanensis*
22. Pileus with soft, more or less erect conical to pyramidal scales; stipe without an annular zone, entirely with black granular scales; echinate basidiospores with confluent tubercles and irregular incomplete reticulations*S*. *calidus*22. Pileus with hard, more or less erect conical scales; stipe with an annular zone at apex, with black minutely conical scales and fluffy flosses; basidiospores with semireticulate ornamentation*S*. *giganteus*23. Pileus larger (up to 6 cm); stipe with patch-like to appressed, blackish-brown scales or reticula; tropical distribution*S*. *baozhengii*23. Pileus smaller (up to 4.5 cm); stipe with black tomentose scales; subtropical distribution*S*. *huangshanensis*24. Pileus with more or less erect conical to pyramidal scales2524. Pileus with patch-like to appressed scales or flosses2925. Basidiospores with complete reticula2625. Basidiospores with incomplete reticula2826. Stipe without an annular zone; tropical to subtropical distribution
*S. montosus*
26. Stipe with an annular zone; subtropical to temperate distribution2727. Pileus larger (up to 11 cm), with dirty white, small, erect conical scales and greyish-black apex; hymenophoral pores larger, 0.1–0.2 cm diameter
*S. pteroreticulosporus*
27. Pileus smaller (up to 7 cm), with greyish-black, more or less erect pyramidal scales; hymenophoral pores smaller, 0.05–0.1 cm diameter*S*. *pinophilus*28. Pileus larger (up to 12 cm); stipe with greyish-white flosses on the upper part and black on the lower; echinate basidiospores with irregular short ribs; subtropical to temperate distribution*S*. *densisquamosus*28. Pileus smaller (up to 6 cm); stipe with black scattered granular scales; semireticulate basidiospores*S*. *velutinus*29. Basidiospores with complete reticula3029. Basidiospores with incomplete reticula3130. Pileus larger (up to 15 cm); stipe with an annulus, with grey to dirty white flosses on the upper part and greyish-black on the lower; basidiospores with larger meshes (2–4 μm diameter); subtropical distribution*S*. *glabriceps*30. Pileus smaller (up to 9 cm); stipe without an annular zone, entirely with whitish thick fluffy flosses; basidiospores with smaller meshes (1–2 μm diameter); tropical and subtropical distribution*S*. *latirimosus*31. Stipe with an annular zone; basidiospores smaller measuring 7–9 × 6.5–8.5 μm; tropical to subtropical distribution*S*. *seminudus*31. Stipe without an annular zone; basidiospores larger measuring 8–10 × 7.5–8.5 μm; subtropical distribution*S*. *subnudus*


## Figures and Tables

**Table 1:** Taxa, vouchers, locations, and GenBank accession numbers of DNA sequences used in phylogenetic analyses. The new sequences are in bold.

Taxon	Voucher	Locality	GenBank Accession No.				Reference
			28S	ITS	*tef*1	*rpb*2	
*Afroboletus luteolus*	PC0723573	Zambia	—	—	KX869290	KX869416	[5]
*Afroboletus luteolus*	PC0723568	Madagascar	—	—	KX869291	KX869417	[5]
*Afroboletus multijugus*	PC0723570	Burundi	—	—	KX869298	KX869425	[5]
*Afroboletus multijugus*	PC0723571	Burundi	—	—	KX869299	KX869426	[5]
*Afroboletus multijugus*	PC0723580	Madagascar	—	—	KX869300	KX869427	[5]
*Anthracoporus holophaeus*	HKAS50508	Yunnan, SW China	KF112465	—	KX869376	KX869506	[5,19]
*Anthracoporus holophaeus*	HKAS59407	Yunnan, SW China	KT990708	—	KT990888	KT990506	[20]
*Strobilomyces* aff. *verruculosus*	HKAS55389	China	KF112461	—	KF112259	KF112813	[19]
*Strobilomyces albidus*	HKAS89104 (holotype)	Yunnan, SW China	—	—	KX869340	KX869469	[5]
*Strobilomyces albidus*	NY1393538	Thailand	—	—	KX869343	KX869472	[5]
*Strobilomyces alpinus*	HKAS77969	Hubei, central China	—	MG832045	KX869254	KX869380	[5,7]
*Strobilomyces alpinus*	HKAS57770	Yunnan, SW China	—	MG832044	—	KX869382	[5,7]
*Strobilomyces alpinus*	HKAS94051	Tibet, SW China	—	—	KX869255	KX869381	[5]
*Strobilomyces annulatus*	NY1393525	Papua New Guinea	—	—	KX869256	KX869383	[5]
*Strobilomyces anthracinus*	HKAS74532	Yunnan, SW China	—	MG832047	KX869330	KX869459	[5,7]
*Strobilomyces anthracinus*	HKAS52570	Yunnan, SW China	—	—	KX869331	KX869460	[5]
*Strobilomyces anthracinus*	HKAS83740 (holotype)	Yunnan, SW China	—	—	KX869329	KX869458	[5]
*Strobilomyces atrosquamosus*	HKAS78563	Yunnan, SW China	KT990649	MG832048	KX869262	KX869388	[5,7,20]
*Strobilomyces atrosquamosus*	HMAS262149	Guizhou, SW China	—	—	KX869259	KX869385	[5]
*Strobilomyces atrosquamosus*	HKAS84682	Yunnan, SW China	—	MG832049	KX869261	KX869387	[5,7]
***Strobilomyces baozhengii***	**N.K. Zeng2976 (FHMU1937)** **(holotype)**	**Hainan, southern China**	**OR036230**	**OR035750**	**OR051644**	**OR051645**	**Present study**
***Strobilomyces baozhengii***	**N.K. Zeng3341 (FHMU3129)**	**Hainan, southern China**	**—**	**OR035751**	**—**	**OR051646**	**Present study**
*Strobilomyces brunneolepidotus*	HKAS81935	Fujian, SE China	—	MG832053	KX869265	KX869391	[5,7]
*Strobilomyces brunneolepidotus*	HKAS84683	Yunnan, SW China	—	—	KX869264	KX869390	[5]
*Strobilomyces brunneolepidotus*	HKAS80689	Guangdong, southern China	—	—	KX869263	KX869389	[5]
*Strobilomyces brunneolepidotus*	N.K. Zeng2575 (FHMU1682)	Hainan, southern China	MT829135	MT822947	—	—	Unpublished
*Strobilomyces calidus*	HKAS87084	Yunnan, SW China	—	MG832085	KX869358	KX869487	[5,7]
*Strobilomyces calidus*	HKAS84700 (holotype)	Yunnan, SW China	—	MG832084	KX869359	KX869488	[5,7]
*Strobilomyces cingulatus*	HKAS75582	Hubei, central China	—	MG832051	KX869334	KX869463	[5,7]
*Strobilomyces cingulatus*	HKAS73175 (holotype)	Yunnan, SW China	—	MG832050	KX869332	KX869461	[5,7]
*Strobilomyces cingulatus*	HKAS91330	Jilin, NE China	—	—	KX869335	KX869464	[5]
*Strobilomyces confusus*	WU17032	USA	—	MG832054	KX869267	KX869393	[5,7]
*Strobilomyces confusus*	WU17130	USA	—	—	KX869268	KX869394	[5]
*Strobilomyces confusus*	CFMR DR-3024	Dominican Republic	MK601809	—	MK721163	MK766365	[33]
***Strobilomyces conicus***	**N.K. Zeng3116 (FHMU2077) (holotype)**	**Hainan, southern China**	**OR036231**	**OR035752**	**OR051632**	**—**	**Present study**
*Strobilomyces densisquamosus*	HKAS91250	Liaoning, NE China	—	—	KX869357	KX869486	[5]
*Strobilomyces densisquamosus*	HKAS82354	Yunnan, SW China	—	MG832055	KX869352	KX869481	[5,7]
*Strobilomyces densisquamosus*	HKAS83112 (holotype)	Yunnan, SW China	—	—	KX869354	KX869483	[5]
*Strobilomyces douformis*	HKAS87097 (holotype)	Yunnan, SW China	—	MG832058	KX869345	KX869474	[5,7]
*Strobilomyces douformis*	HKAS87134	Yunnan, SW China	—	MG832057	KX869346	KX869475	[5,7]
*Strobilomyces echinatus*	Sydney778	Congo	—	—	KX869271	KX869397	[5]
*Strobilomyces echinatus*	PC0723576	Burundi	—	—	KX869270	KX869396	[5]
*Strobilomyces echinatus*	Sydney1597	Congo	—	—	KX869269	KX869395	[5]
*Strobilomyces echinocephalus*	TO MG001 (holotype)	Yunnan, SW China	—	JX870649	—	—	[8]
*Strobilomyces echinocephalus*	HKAS59420	Yunnan, SW China	KF112463	MG832059	KX869274	KX869400	[5,7,19]
*Strobilomyces echinocephalus*	HKAS92153	Hubei, central China	—	—	KX869275	KX869401	[5]
*Strobilomyces floccopus*	Sf1	Germany	DQ534626	—	JQ327037	—	[34,35]
*Strobilomyces floccopus*	AFTOL-ID 716	—	AY684155	AY854068	AY883428	AY786065	Unpublished
*Strobilomyces floccopus*	RW103	Belgium	—	—	KT824044	KT824011	[36]
*Strobilomyces foveatus*	FRI62957	Malaysia	—	—	KX869277	KX869403	[5]
*Strobilomyces foveatus*	FRI69468	Malaysia	—	—	KX869276	KX869402	[5]
*Strobilomyces giganteus*	HKAS93250	Guangdong, southern China	—	MG832062	—	KX869454	[5,7]
*Strobilomyces glabellus*	HKAS74887	Yunnan, SW China	—	MG832063	KX869278	KX869404	[5,7]
*Strobilomyces glabriceps*	HKAS78573	Yunnan, SW China	—	MG832065	KX869281	KX869407	[5,7]
*Strobilomyces glabriceps*	HKAS74964	Yunnan, SW China	—	MG832064	KX869280	KX869406	[5,7]
*Strobilomyces glabriceps*	SharmaS357	India	—	—	KX869279	KX869405	[5]
***Strobilomyces hainanensis***	**N.K. Zeng3210 (FHMU2171) (holotype)**	**Hainan, southern China**	**OR036232**	**—**	**OR051633**	**—**	**Present study**
*Strobilomyces huangshanensis*	HKAS:102613 (holotype)	Anhui, eastern China	—	—	MK329219	MK329217	[37]
*Strobilomyces huangshanensis*	HKAS:102612	Anhui, eastern China	—	—	MK329218	MK329216	[37]
*Strobilomyces latirimosus*	HKAS74865	Yunnan, SW China	KF112467	MG832066	KX869284	KX869410	[5,7,19]
*Strobilomyces latirimosus*	HKAS53348	Fujian, SE China	KF112462	—	KX869289	KX869415	[5,19]
*Strobilomyces latirimosus*	HKAS77697	Guangdong, southern China	—	—	KX869288	KX869414	[5]
*Strobilomyces latirimosus*	HMAS250943	Guizhou, SW China	—	—	KX869287	KX869413	[5]
“*Strobilomyces longistipitatus*”	DC 15-10	India	KX364695	KX364694	—	—	Unpublished
“*Strobilomyces longistipitatus*”	SUB828	Pakistan	MK518065	MK518063	—	—	[38]
*Strobilomyces microreticulatus*	HKAS74863 (holotype)	Yunnan, SW China	—	MG832069	KX869337	KX869466	[5,7]
*Strobilomyces microreticulatus*	HKAS74963	Yunnan, SW China	—	MG832068	KX869336	KX869465	[5,7]
*Strobilomyces minor*	HKAS:101909 (holotype)	Hunan, central China	—	—	MK183829	MK183827	[21]
*Strobilomyces minor*	HKAS:101910	Hunan, central China	—	—	MK183830	MK183828	[21]
*Strobilomyces mirandus*	HKAS80364	Fujian, SE China	—	MG832071	—	KX869420	[5,7]
*Strobilomyces mirandus*	HKAS59408	Yunnan, SW China	—	MG832070	KX869293	KX869419	[5,7]
*Strobilomyces mirandus*	OR115	Thailand	—	—	KT824038	KT824005	[36]
*Strobilomyces mollis*	HKAS59833	Hainan, southern China	—	—	KX869295	KX869422	[5]
*Strobilomyces montosus*	HKAS74910	Yunnan, SW China	—	MG832072	KX869297	KX869424	[5,7]
*Strobilomyces montosus*	HKAS74809	Yunnan, SW China	KF112459	—	KX869296	KX869423	[5,19]
***Strobilomyces pachycystidiatus***	**N.K. Zeng2987 (FHMU1948) (holotype)**	**Hainan, southern China**	**OR036233**	**OR035753**	**OR051634**	**OR051647**	**Present study**
***Strobilomyces pachycystidiatus***	**N.K. Zeng2248 (FHMU1493)**	**Hainan, southern China**	**OR036234**	**OR035754**	**OR051635**	**OR051648**	**Present study**
***Strobilomyces pachycystidiatus***	**N.K. Zeng2937 (FHMU1907)**	**Hainan, southern China**	**OR036235**	**OR035756**	**OR051636**	**—**	**Present study**
***Strobilomyces pachycystidiatus***	**N.K. Zeng4427** **(FHMU4706)**	**Hainan, southern China**	**OR036236**	**OR035757**	**OR051637**	**—**	**Present study**
***Strobilomyces pachycystidiatus***	**N.K. Zeng4431** **(FHMU4666)**	**Hainan, southern China**	**OR036237**	**OR035758**	**OR051638**	**—**	**Present study**
***Strobilomyces pachycystidiatus***	**N.K. Zeng4434** **(FHMU4692)**	**Hainan, southern China**	**OR036238**	**OR035759**	**OR051639**	**—**	**Present study**
***Strobilomyces pachycystidiatus***	**N.K. Zeng2939** **(FHMU1909)**	**Hainan, southern China**	**OR036239**	**OR035760**	**OR051640**	**OR051649**	**Present study**
***Strobilomyces pachycystidiatus***	**N.K. Zeng4469** **(FHMU4751)**	**Hainan, southern China**	**OR036240**	**OR035761**	**OR051641**	**OR051650**	**Present study**
***Strobilomyces pachycystidiatus***	**N.K. Zeng2930 (FHMU1900)**	**Hainan, southern China**	**OR036241**	**OR035762**	**OR051642**	**—**	**Present study**
*Strobilomyces parvirimosus*	HKAS74547	Yunnan, SW China	—	MG832073	KX869302	KX869429	[5,7]
*Strobilomyces parvirimosus*	HKAS74911	Yunnan, SW China	KF112460	—	KX869301	KX869428	[5,19]
*Strobilomyces pinophilus*	HKAS80300 (holotype)	Anhui, eastern China	—	MG832074	KX869350	KX869479	[5,7]
*Strobilomyces pinophilus*	HKAS94132	Shandong, eastern China	—	—	KX869351	KX869480	[5]
*Strobilomyces pteroreticulosporus*	HKAS80299	Anhui, eastern China	—	MG832076	KX869304	KX869431	[5,7]
*Strobilomyces pteroreticulosporus*	HKAS80350	Shaanxi, NW China	—	MG832075	KX869303	KX869430	[5,7]
*Strobilomyces pteroreticulosporus*	HKAS91274	Liaoning, NE China	—	—	KX869306	KX869433	[5]
*Strobilomyces pteroreticulosporus*	HKAS80191	Hubei, central China	—	—	KX869305	KX869432	[5]
*Strobilomyces rubrobrunneus*	HKAS:101906 (holotype)	Anhui, eastern China	—	—	MH485372	MH485369	[6]
*Strobilomyces sculptus*	HKAS:102606	Anhui, eastern China	—	—	MK241491	MK241488	[22]
*Strobilomyces sculptus*	HKAS:101911	Anhui, eastern China	—	—	MK241490	MK241487	[22]
*Strobilomyces sculptus*	HKAS:102610 (holotype)	Anhui, eastern China	—	—	MK241489	MK241486	[22]
*Strobilomyces seminudus*	HKAS59461	Yunnan, SW China	KF112479	—	KX869311	KX869438	[5,19]
*Strobilomyces seminudus*	NY1393550	Thailand	—	—	KX869310	KX869437	[5]
*Strobilomyces seminudus*	HKAS77085	Jiangxi, eastern China	—	—	KX869309	KX869436	[5]
*Strobilomyces seminudus*	HKAS83471	Sichuan, SW China	—	—	KX869307	KX869434	[5]
*Strobilomyces seminudus*	HKAS82848	Chongqing, SW China	—	—	KT990835	KT990472	[20]
*Strobilomyces* sp.	HKAS84055	USA	—	MG832083	KX869366	KX869495	[5,7]
*Strobilomyces* sp.	WU17052	Mexico	—	MG832082	KX869338	KX869467	[5,7]
*Strobilomyces* sp.	WU17033	Mexico	—	MG832081	KX869339	KX869468	[5,7]
*Strobilomyces* sp.	WU17057	USA	—	MG832080	KX869365	KX869494	[5,7]
*Strobilomyces* sp.	MEL695356	Australia	—	—	KX869370	KX869499	[5]
*Strobilomyces* sp.	HKAS92326	Hubei, central China	—	—	KX869369	KX869498	[5]
*Strobilomyces* sp.	PC0723581	Madagascar	—	—	KX869368	KX869497	[5]
*Strobilomyces* sp.	NY1393513	Australia	—	—	KX869367	KX869496	[5]
*Strobilomyces* sp.	HKAS74893	Yunnan, SW China	—	—	KX869364	KX869493	[5]
*Strobilomyces* sp.	NY1193833	Australia	—	—	KX869361	KX869490	[5]
*Strobilomyces* sp.	NY1193834	Australia	—	—	KX869360	KX869489	[5]
*Strobilomyces* sp.	NY2072541	Australia	—	—	KX869349	KX869478	[5]
*Strobilomyces* sp.	NY1034408	Australia	—	—	KX869348	KX869477	[5]
*Strobilomyces* sp.	NY1491183	Australia	—	—	KX869347	KX869476	[5]
*Strobilomyces* sp.	NY1034411	Australia	—	—	KX869325	KX869452	[5]
*Strobilomyces* sp.	NY1034410	Australia	—	—	KX869324	KX869451	[5]
*Strobilomyces* sp.	CFMR DR-555	Dominican Republic	MK601810	—	MK721164	MK766366	[33]
*Strobilomyces* sp.	JLF6840	USA	MN294430	MN306149	—	—	Unpublished
*Strobilomyces* sp.	JLF6808	USA	MN294429	MN306148	—	—	Unpublished
*Strobilomyces* sp.	OR1092	Thailand	—	—	MH614739	MH614786	[39]
*Strobilomyces* sp.	OR0319	Thailand	—	—	MH614738	MH614785	[39]
*Strobilomyces* sp.	OR0778	Thailand	—	—	MG212610	MG212651	[40]
*Strobilomyces* sp.	OR0259	China	—	—	MG212609	MG212650	[40]
*Strobilomyces* sp.	NY0075256	—	—	KM460929	—	KM460933	Unpublished
“*Strobilomyces strobilaceus*”	HKAS95079	Yunnan, SW China	—	—	KX869317	KX869444	[5]
“*Strobilomyces strobilaceus*”	HKAS75466	Hubei, central China	KT990644	—	KX869315	KX869442	[5,20]
*Strobilomyces strobilaceus*	WU17111	Mexico	—	MG832086	KX869316	KX869443	[5,7]
*Strobilomyces strobilaceus*	WU0016537	Austria	KT990647	—	KX869314	KX869441	[5,20]
*Strobilomyces strobilaceus*	MB001177	Germany	—	—	KX869313	KX869440	[5]
*Strobilomyces subnudus*	HKAS59435	Yunnan, SW China	KF112464	MG832088	KX869318	KX869445	[5,7,19]
*Strobilomyces subnudus*	HKAS82418	Taiwan, SE China	—	MG832087	KX869321	KX869448	[5,7]
*Strobilomyces subnudus*	HKAS84811	Yunnan, SW China	—	—	KX869320	KX869447	[5]
*Strobilomyces velutinus*	HKAS84755	Yunnan, SW China	—	MG832089	KX869322	KX869449	[5,7]
*Strobilomyces velutinus*	HKAS84776	Yunnan, SW China	—	—	KX869323	KX869450	[5]
*Strobilomyces velutipes*	NY1193952	Australia	—	—	KX869363	KX869492	[5]
*Strobilomyces velutipes*	NY2072516	Australia	—	—	KX869362	KX869491	[5]
“*Strobilomyces verruculosus*”	HKAS77026	Jiangxi, eastern China	—	—	KX869328	KX869456	[5]
“*Strobilomyces verruculosus*”	NY1393514	Thailand	—	—	KX869327	KX869455	[5]
“*Strobilomyces verruculosus*”	HKAS59637	Yunnan, SW China	KT990645	—	KT990838	KT990475	[20]

SE = southeastern, SW = southwestern, NE = northeastern, NW = northwestern.

**Table 2:** Accepted species, and locality of *Strobilomyces* (sect. *Strobilomyces*) in China.

Species	Locality	References
*Strobilomyces albidus*	Yunnan, SW China	[7]
*Strobilomyces alpinus*	Yunnan, SW China	[12]
*Strobilomyces anthracinus*	Yunnan, SW China	[7]
*Strobilomyces atrosquamosus*	Yunnan, SW China	[46]
*Strobilomyces baozhengii*	Hainan, southern China	**Present study**
*Strobilomyces brunneolepidotus*	Japan	[44]
*Strobilomyces calidus*	Yunnan, SW China	[7]
*Strobilomyces cingulatus*	Yunnan, SW China	[7]
*Strobilomyces conicus*	Hainan, southern China	**Present study**
*Strobilomyces densisquamosus*	Yunnan, SW China	[7]
*Strobilomyces douformis*	Yunnan, SW China	[7]
*Strobilomyces echinocephalus*	Yunnan, SW China	[8]
*Strobilomyces giganteus*	Sichuan, SW China	[12]
*Strobilomyces glabellus*	Yunnan, SW China	[11]
*Strobilomyces glabriceps*	Yunnan, SW China	[47]
*Strobilomyces hainanensis*	Hainan, southern China	**Present study**
*Strobilomyces huangshanensis*	Anhui, eastern China	[37]
*Strobilomyces latirimosus*	Guangxi, southern China	[11]
*Strobilomyces microreticulatus*	Yunnan, SW China	[7]
*Strobilomyces minor*	Hunan, central China	[21]
*Strobilomyces mirandus*	Malaysia	[1]
*Strobilomyces mollis*	Malaysia	[1]
*Strobilomyces montosus*	India	[42]
*Strobilomyces pachycystidiatus*	Hainan, southern China	**Present study**
*Strobilomyces parvirimosus*	Yunnan, SW China	[42]
*Strobilomyces pinophilus*	Anhui, eastern China	[7]
*Strobilomyces pteroreticulosporus*	Korea	[15]
*Strobilomyces rubrobrunneus*	Anhui, eastern China	[6]
*Strobilomyces sculptus*	Anhui, eastern China	[22]
*Strobilomyces seminudus*	Japan	[48]
*Strobilomyces subnudus*	Jiangsu, eastern China	[11]
*Strobilomyces velutinus*	Yunnan, SW China	[11]

SW = southwestern.

## Data Availability

The names of the new species were formally registered in the MycoBank [https://www.mycobank.org/ (accessed on 1 October 2023)]. Specimens were deposited in the Fungal Herbarium of Hainan Medical University, Haikou City, Hainan Province of China (FHMU). The sequence data generated in this study are deposited in GenBank [https://www.ncbi.nlm.nih.gov/genbank/ (accessed on 30 June 2023)].
